# Suppression of *Magnaporthe oryzae* and interaction between *Bacillus subtilis* and rice plants in the control of rice blast

**DOI:** 10.1186/s40064-016-2858-1

**Published:** 2016-08-02

**Authors:** Yuexia Sha, Qi Wang, Yan Li

**Affiliations:** 1 Department of Plant Pathology, China Agricultural University, 2 West Yuanmingyuan Rd., Haidian District, Beijing, 100193 China; 2 Key Laboratory of Plant Pathology, Ministry of Agriculture, China Agricultural University, Beijing, 100193 China

**Keywords:** *Bacillus subtilis*, *Magnaporthe oryzae*, Rice plant, Colonization, Antifungal activity, Defense enzymes

## Abstract

*Magnaporthe oryzae*, the causative pathogen of rice blast, has caused extensive losses to rice cultivation worldwide. Strains of the bacterium *Bacillus subtilis* have been used as biocontrol agents against rice blast. However, little has been reported about the interaction between *B. subtilis* and the rice plant and its mechanism of action. Here, the colonization process and induced disease resistance by *B. subtilis* SYX04 and SYX20 in rice plants was examined. Strains of *B. subtilis* labeled with green fluorescent protein reached population of more than 5 × 10^6^ CFU/g after 20 days on mature rice leaves and were detected after 3 days on newly grown leaves. Results showed that SYX04 and SYX20 not only inhibited spore germination, germ tube length, and appressorial formation but also caused a series of alterations in the structures of hyphae and conidia. The cell walls and membrane structures of the fungus showed ultrastructural abnormalities, which became severely degraded as observed through scanning electron microscopy and transmission electron microscopy. The mixture of both *B. subtilis* and *M. oryzae* resulted in enhanced activity of peroxidase, and polyphenol oxidase while there was significantly more superoxide dismutase activity in plants that had been sprayed with *B.subtilis* alone. The present study suggests that colonized SYX04 and SYX20 strains protected rice plants and exhibited antifungal activity and induced systemic resistance, thus indicating their potential biological control agents.

## Background

Rice (*Oryza sativa*), an important staple food crop, is cultivated on over 160 million hectares worldwide. It provides the daily energy for over 3.5 billion people (Skamnioti and Gurr 2009; FAO 2012; Muthayya et al. 2014). Rice blast caused by *Magnaporthe oryzae*, spread in more than 85 countries and has caused great yield loss. Some 30 % of the annual rice harvest is lost due to rice blast infection, enough rice to feed more than 60 million people for 1 year (Dagdas et al. 2012). Chemical pesticides, though effective in blast control (Yamaguchi 2004), involve residual toxicity and environmental pollution. Unlike applications of chemical fungicides, the use of biocontrol bacteria can involve mixtures of antifungal compounds, produced by the bacterium in amounts that fluctuate based on environmental cues (Hoitink and Boehm 1999).

Interactions between biocontrol agents and pathogens have been studied in rice (Zarandi et al. 2009) and other commercially important crops (Anand et al. 2010). Yan et al. (2015) studied the interactions between fungi and bacteria in rice and found that *Burkholderia gladioli* strain could have a competitive superiority with *Aspergillus flavus* strain. Kumar et al. (2013) analyzed interactions between *Bacillus subtilis* MBI 600 and *Rhizoctonia solaniplanta* and found that strain MBI 600 suppressed its fungal partner effectively. The present study evaluated interactions between antagonistic bacteria and phytopathogens. Results showed *Pseudomonas fluorescens* 2137 to be highly effective in suppressing *Fusarium culmoru*m on barley roots (Strunnikova et al. 2015). The genus *Bacillus* has considerable possible uses in agriculture. The antifungal properties of *Bacillus* species, including *B.* *subtilis*, have been investigated for their importance to the biological control of a number of plant and animal diseases (Grover et al. 2010). In addition, beneficial effects conferred by *Bacillus* strains act directly through several different biocontrol mechanisms, such as colonizing plants, forming biofilms, and enhancing the provision of nutrients and phytohormones, though they can also rely on other mechanisms, including antifungal activity and induced systemic resistance. A few studies have investigated the impact of *B.* *subtilis*. The colonization of *B. megaterium* (Liu et al. 2006) and strains of *B. methylotrophicus* (Shan et al. 2013) in rice plants has also been reported to have antifungal activity and to induce systemic resistance towards rice blast. In another study, higher levels of the PR proteins (PR2, PR6, PR15, and PR16) were observed in rice leaves inoculated with *B. subtilis* CB-R05 compared with the untreated control, indicating their potential for use in the biological control of the disease (Ji et al. 2014).

It is vitally important to develop successful and lasting solutions to crop loss caused by plant diseases. Understanding the global effect of biocontrol bacteria on phytopathogens may greatly increase the size and safety of the global food supply. In previous studies, several bacteria were isolated from rice cultivar Rejingyou No. 35 from Southwest Territories of China, which not only improved plant growth, but also suppressed *M.* *oryzae*. *B. subtilis* strains SYX04 and SYX20 have been characterized, and their antifungal activities against indicator fungi pathogen *M. oryzae* P131 have been demonstrated and found to be efficient. In a 2-year field experiment performed at several different locations, the use of *B. subtilis* SYX04 and SYX20 reduced the incidence of leaf blast by 73.5–83.5 % and that of panicle blast by 64.0–85.6 % (Sha et al. 2014). This effect was similar to the use of 75 % tricyclazole wettable powder. These strains have considerable prospects for use as a new tool for the biocontrol of rice blast. The purpose of the present study was to clarify bio-control mechanisms of *B.* *subtilis* against *M.* *oryzae* P131. To our knowledge, this is the first detailed report of the interaction between GFP-tagged *B.* *subtilis* and rice plant was performed towards the control of rice blast.

## Methods

### Bacterial strains and plasmids

*Bacillus subtilis* SYX04 and SYX20 (GenBank Accession Number: KJ848470 and KJ84847) were isolated from rice leaves at the Key Laboratory of Plant Pathology, China Agricultural University. The shuttle vector pGFP78 for *Escherichia coli*–*B. subtilis* containing GFP (Dh5α) and tetracycline (50 μg/mL) resistance genes (Dunn and Handelsman 1999; Tian et al. 2004) was used as described by Li et al. (2000). Construction of GFP-labeled *B.* *subtilis* SYX04 and SYX20 were prepared as previously described (Li et al. 2000).

### Co-culture assays of *B. subtilis* and *M. oryzae* P131

*Bacillus subtilis* was cultured in LB broth (300 mL/L in a liter flask) until OD600 values reached 1.0–1.2. Then liquid culture was centrifuged at 15 kg for 15 min at 4 °C and filtered by 0.22 µm biofilter for culture filtrate. *M.* *oryzae* P131 was incubated on tomato-oat medium for 9 days. Then, the petri dishes were washed twice with sterile water for spore suspension. The co-culture experiments were carried out in liquid culture of *B.* *subtilis* SYX04 or SYX20 (1 × 10^8^ CFU/mL) and *M.* *oryzae* P131 spore suspension (1 × 10^6^ cell/mL) or culture filtrate and *M.* *oryzae* P131 spore suspension in the same shake-flasks at 28 °C at a 1:1 volume ratio. The co-culture samples were incubated for 4, 8, 12, and 24 h in culture conditions as described previously. All procedures described above were carried out under sterile conditions.

### Population dynamics of GFP-tagged SYX04 and SYX20

Rice cv. Lijiangxintuanheigu (LTH) was used as a host plant (Kobayashi et al. 2007) susceptible to *M. oryzae* P131. Rice seeds were surface-sterilized by 1 % sodium hypochlorite solution for 20 min and 75 % ethanol for 10 min and then washed 3 times with sterile water. Finally, rice seeds that had been soaked in sterile water were germinated in a sterile petri dish for 2 days at 28 °C in a growth cabinet. When rice seeds reached approximately 1 cm in length, all seedlings were planted in sterile soil buckets and cultured in greenhouse. At the rice tillering stage (planted about 60 days), the plants were sprayed with GFP-labeled *B. subtilis* suspensions (1 × 10^8^ CFU/mL) or water inoculation for the negative controls. Each treatment group included twelve plants. Each bucket had three rice plants and each bucket was sprayed with 50 mL suspensions. The colonization dynamics was tested on leaves at 1, 3, 5, 7, 14, and 20 days post-transplant. Rice leaves were weighed (fresh weight 1 g), then grinded in a sterile mortar with 4 mL sterile water and a small amount of sterile quartz sands. Finally, samples were grounded in a conical flask with 96 mL sterile water and allowed to be incubated with shaking for 30 min, and the supernatant was diluted to 1:10^3^ and spread on a petri dish.

### Impact of *B. subtilis* on fungal cell

The following procedure was performed to determine the inhibitive effect of *B.* *subtilis* on fungal spore germination, germ length, and appressorial formation. Spore germination and morphological changes to the co-culture samples were monitored microscopically using centrifuge tubes. These co-culture samples were observed under an Olympus DP71 microscope. Images were captured using an Olympus digital camera, exported as TIFF files, and adjusted with Corel PHOTO-PAINT X6 software (Corel Corp, Ottawa, Ontario, Canada). All experiments were performed in triplicate. The relative number of germinated conidia, germ tube length, and appressorial formation were investigated by light microscopic examination of at least 100 spores per replicate (Zhang et al. 2013). Inhibition of spore germination, germ tube formation, and appressorial formation were calculated from the test data as follows:

$${\text{Inhibition}}\,(\% ) = 100 \times (A - B)/A$$

where *A* and *B* are the relative numbers of germinated spores, germ tubes, and appressoria in the control and test samples, respectively.

### SEM and TEM examinations

For scanning electron microscopy (SEM) examination, the co-culture samples that blended *B.* *subtilis* SYX04 and SYX20 cells (1 × 10^8^ CFU/mL) and spore suspension of *M.* *oryzae* P131 (1 × 10^6^ CFU/mL) by the same volume were incubated for 12 h in culture conditions as described previously, and *M.* *oryzae* P131 without *B.* *subtilis* cells served as a control. The conidial and hyphal structures of *M.* *oryzae* P131 inhibited by *B.* *subtilis* SYX04 and SYX20 cells were fixed in 4 % glutaraldehyde at 4 °C for 16 h, dehydrated, and coated with gold as described previously (Kong et al. 2012). The samples were observed with an S-3400N (Hitachi, Tokyo, Japan) scanning electron microscope as previously described (Kong et al. 2012). For transmission electron microscopic (TEM) examination, the co-culture samples that contained both *B.* *subtilis* SYX04 or SYX20 cells (1 × 10^8^ CFU/mL) and spore suspension of *M. oryzae* P131 (1 × 10^6^ CFU/mL) at the same volume were incubated for 4, 8, 12, and 24 h in culture conditions as described previously, and the co-cultures without *B. subtilis* cells served as a controls. The conidial and hyphal structures of *M.* *oryzae* P131 were fixed, dehydrated, and embedded as previously described (Mariné 1992). The samples were made into ultrathin sections approximately 60 nm thick with an ultramicrotome (UC-7; Lecia Inc., Wetzslar, Germany). Serial thin-sections were removed onto slot grids and observed with a JEM-1230 transmission electron microscope (JEOL Ltd., Tokyo, Japan).

### Assay of defense enzymes

At 20 days after growing in a plant growth cabinet at 28 °C and 12 h light per day, rice plants were sprayed with 1 × 10^8^ CFU/mL bacterial cells and 1 × 10^6^ CFU/mL suspensions of *M.* *oryzae* P131. Rice leaf samples were collected at different times after inoculation (1, 2, 3, 4, 5, and 6 days). Each replication had six plants sprayed with bacterial cells, or suspensions of *M.* *oryzae* P131, or mixture of bacterial cells and suspensions of *M.* *oryzae* P131. The experiments were performed using completely randomized designs, six treatments, and four replicates per treatment. The experimental treatments were as follows: treatment with a mixture of fermentation broth of SYX04 and pathogen infection (BP); treatment with a mixture of fermentation broth of SYX20 and pathogen infection (MP); treatment with a fermentation broth control of SYX04 (BC); treatment with a fermentation broth control of SYX20 (MC); treatment with a *M.* *oryzae* P131 control (PC); and treatment with sterile water control (UC). Peroxidase (PO) activity was detected as described by Hammerschimidt et al. (1982), by measuring absorbance at 470 nm. Polyphenol oxidase (PPO) activity was examined as described by Li and Steffens (2002), by measuring absorbance at 420 nm. The changes in absorbance (OD) for PO and PPO measurements were assayed at 30 s intervals for up to 3 min and are expressed in terms of U/g/min of protein. Superoxide dismutase (SOD) activity was examined as described by Dhindsa et al. (1981) by measuring absorbance at 560 nm. One unit of SOD denotes the amount of enzyme that inhibits the NBT photoreduction by 50 %, and the enzyme was quantified based on the relative amount of inhibition. The enzyme activity of SOD is here expressed in terms of unit/g of protein.

### Statistical analysis

All treatments were designed in four replicates. All analysis were carried out by the LSD’s test (*P* ≤ 0.05) following one-way ANOVA using the SPSS 19.0 software for Windows (SPSS Inc., Chicago, IL, US).

## Results

### Population dynamics of *B. subtilis* SYX04 and SYX20 colonization of rice plants

**Fig. 1:**
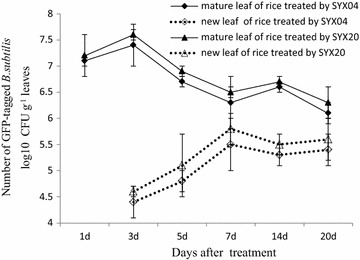
Bacterial cell counts in the rice plants after inoculation with GFP-tagged *B.* *subtilis* SYX04 and SYX20. On 1–2 days after inoculation, rice grew short new leaves and it was difficult to collect leaf samples. Starting from 3 days after inoculation, the colonization of new leaves was counted. The *error bars* indicate the SD of the mean calculated from three independent samples

Almost 1 × 10^8^ CFU/g of rice leaf SYX04 and SYX20 cells were detected immediately after inoculation (Fig. 1). After 24 h, the abundance decreased to 1.3 × 10^7^ CFU/g of leaf. After 72 h, the cell counts in the leaves peaked at about 3 × 10^7^ CFU/g of leaf. At 2 weeks, the bacterial counts had a small peak at about 6.6 × 10^6^ CFU/g after which the cell counts decreased further. More than 5 × 10^6^ CFU/g labeled strains were detected after 20 days in rice mature leaves and labeled strains could be also detected after 3 days in rice newly grown leaves. The colonization of *B. subtilis* SYX04 and SYX20 in rice plants suggests that the infection sites are probably occupied.

### Inhibition of conidial germination and appressorial formation of *M.* *oryzae* by *B.* *subtilis*

The effects of *B.* *subtilis* against *M. oryzae* spore germination, germ tube elongation, and appressorial formation were assayed. Conidial germination and appressorial formation were significantly reduced by all four *B. subtilis* treatments compared to the control. Different treatments affected the germination and appressorial formation of *M.* *oryzae* in different ways (Table 1). The high percentage of inhibition of conidial germination shows that *B.* *subtilis* SYX04 and SYX20 can suppress development of *M. oryzae*.

**Table 1:** Spore germination and germ tube length of *M.* *oryzae* P131 co-cultured with cells or sterile culture filtrate of *Bacillus subtilis* strains SYX04 and SYX20

Treatment	Conidia germination		Appressorial formation		Germ tubes	
	Number	Inhibition (%)	Number	Inhibition (%)	Length (µm)	Inhibition (%)
SYX20 culture	7 ± 0.6 d	93.3 ± 0.6	5 ± 0.3 c	93.8 ± 0.8	12.2 ± 2.2 c	88.3 ± 2.1
SYX20 filtrate	27 ± 1.0 b	72.7 ± 1.0	24 ± 1.0 b	72.1 ± 0.3	27.6 ± 0.6 b	73.5 ± 0.6
SYX04 culture	9 ± 1.2 d	91.3 ± 1.2	6 ± 1.7 c	93.0 ± 2.2	4.3 ± 0.5 d	95.9 ± 0.5
SYX04 filtrate	23 ± 2.9 c	76.8 ± 3.5	22 ± 1.2 b	73.3 ± 1.4	27.8 ± 7.5 b	73.3 ± 7.6
Control	99 ± 0.6 a	–	86 ± 2.0 a	–	104.3 ± 5.4 a	–

SYX20 culture here refers to liquid culture of *B. subtilis* SYX20; SYX20 filtrate refers to culture filtrate of *B. subtilis* SYX20; SYX04 culture refers to liquid culture of *B. subtilis* SYX04; SYX04 filtrate refers to culture filtrate of *B. subtilis* SYX04; control refers to *M. oryzae* P131 alone. Data were first tested for normality and then analyzed using ANOVA. Significant differences between the mean values of each cohort were determined using LSD’s test (*P* = 0.05) following one-way ANOVA. The data is computed at *P* = 0.05 ± SD

Spore germination was found to be significantly inhibited by liquid culture of SYX04 and SYX20, and a similar trend was observed for germ tube length. Samples treated with liquid culture of SYX20 showed a 93.30 and 93.8 % inhibition of spore germination and appressorial formation, respectively. Spore germination and germ tube length were higher in response to culture filtrate containing SYX20, compared to the results for the SYX20 liquid culture. As SYX04 went from liquid culture to culture filtrate, length range of germ tube of *M.* *oryzae* P131 was from 4.3 to 27.8 μm. Liquid culture of SYX04 also inhibited conidial germination and appressorial formation.

The results show an abnormally thick and wide germ tube caused by *B.* *subtilis* SYX20. The defective germ tubes and abnormal appressoria were observed, probably due to abnormal conidia germination caused by *B.* *subtilis* SYX04. The inhibition of germination, reduction in the number of conidia with normal appressoria, and the formation of conidia with defective appressoria, can result in suppression of rice blast.

### Electron microscope analysis of *M. oryzae* P131

**Fig. 2:**
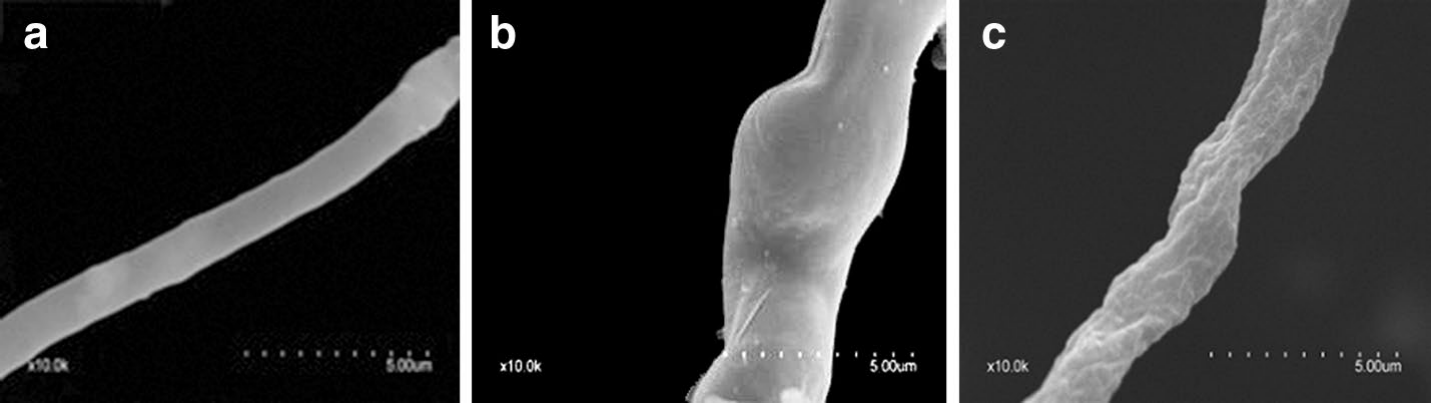
Scanning electron micrographs of *M.* *oryzae*: Hyphae exposed to liquid culture of **a** water, **b** *B.* *subtilis* SYX20, **c** *B. subtilis* SYX04. *Scale bar* 5 μm. Growth inhibition at the hyphal tips was observed in *B. subtilis*-treated fungal cultures. *Scale bar* 10 μm. *Arrows* and *arrowheads* hyphae shrinkage and partial distortion, respectively

The hyphae of *M.* *oryzae* P131 without antagonistic bacteria had a complete tubular shape and were morphologically normal observed by SEM (Fig. 2a). The hyphae treated with *B.* *subtilis* SYX04 culture, showed shrinkage, partial distortion, roughness, and wrinkling of the surface, indicating overall morphological abnormality (Fig. 2b). The hyphae treated with *B.* *subtilis* SYX20 culture displayed swelling and partial distortion (Fig. 2c). The hyphae tips treated with *B.* *subtilis* SYX04 and SYX20 also showed these morphological changes also. The hyphae tips of *M.* *oryzae* P131 without antagonistic bacteria were observed using SEM, which had a finely rounded terminus. After exposure to *B. subtilis* SYX04, the hyphae tips showed tapering and shrinkage, while the hyphae tips treated with *B. subtilis* SYX20 displayed tapering and partial inflation.

**Fig. 3:**
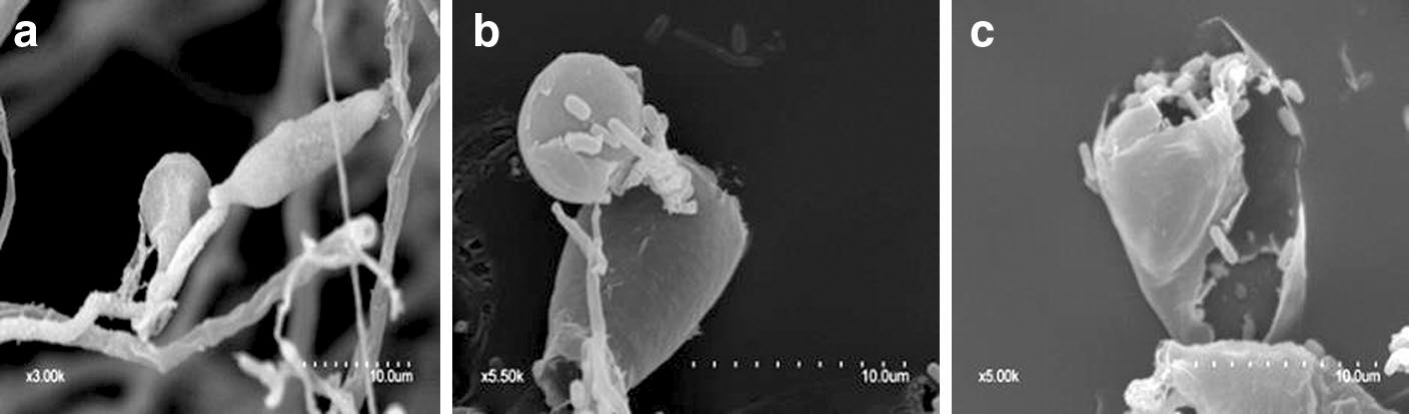
*M. oryzae* conidia grown in the **a** absence of either agent or **b** presence of *B.* *subtilis* SYX20 and **c** *B.* *subtilis* SYX04 at a concentration of 1 × 10^8^ CFU/g. Fungal and liquid culture of *B.* *subtilis* co-cultured at 12 h were examined using a S-3400N scanning electron microscope. *Scale bar* 10 μm

The conidia of *M.* *oryzae* P131 without antagonistic bacteria formed morphologically normal appressoria, as observed under SEM (Fig. 3a). After exposure to *B.* *subtilis* SYX04 and SYX20, the cell wall of conidia appeared to be degraded severely (Fig. 3b, c).

**Fig. 4:**
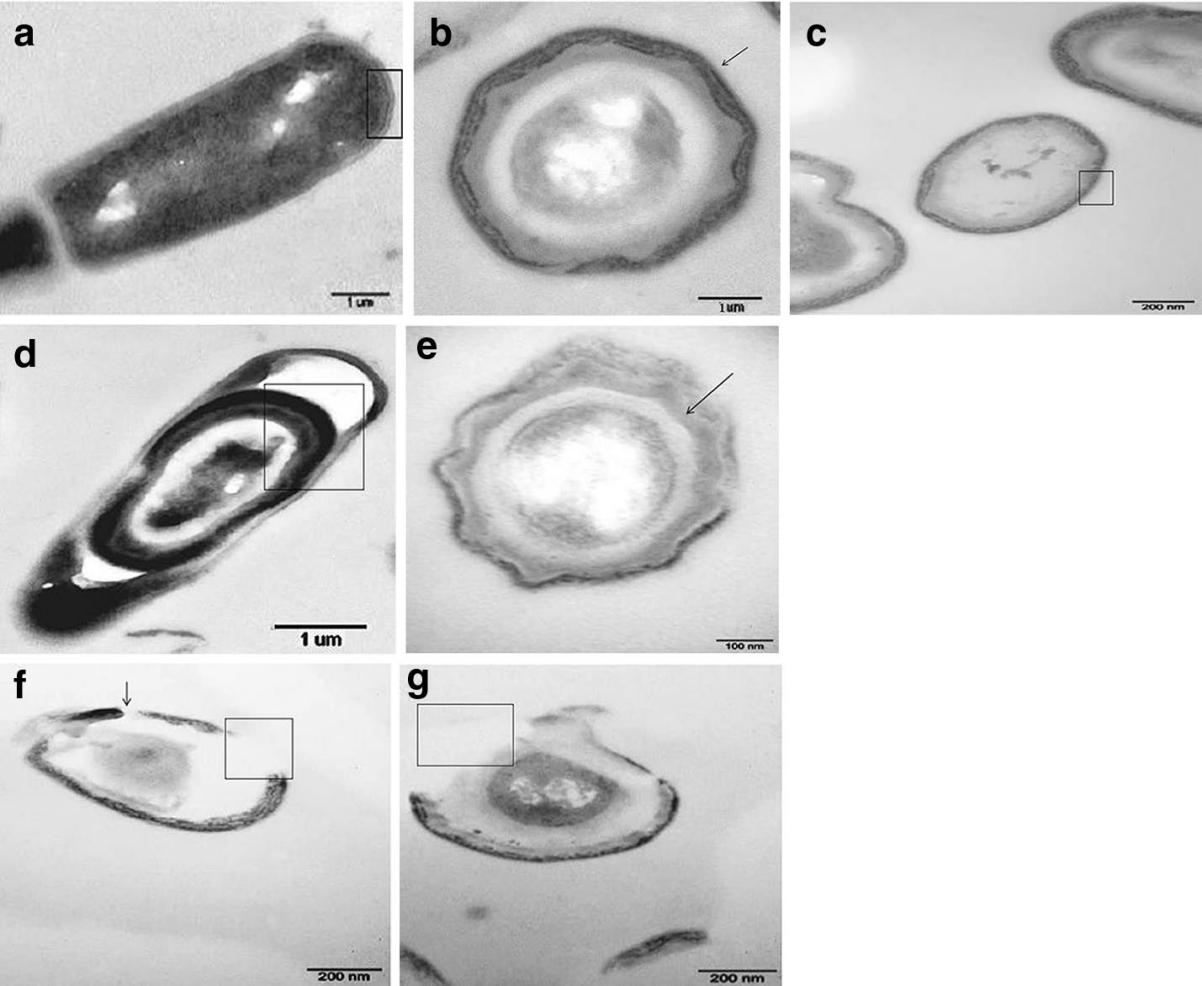
Transmission electron micrographs of *M.* *oryzae* cells exposed to liquid culture of *B.* *subtilis*. *M.* *oryzae* P131 conidia and hyphae in the **a**–**c** absence of either agent, **d**, **e** presence of liquid culture of *B. subtilis* SYX04 and **f**, **g** liquid culture of *B.* *subtilis* SYX20 at a concentration of 1 × 10^8^ CFU/g. Fungal cells treated with SYX04 and SYX20 exhibited significant morphological and ultrastructural abnormalities, such as increased vacuolation, invagination distortion, retraction of the plasma membranes, and formation of polymorphic vesicles. **f**, **g** Loss of plasma membrane integrity and the formation of pores were observed. *Scale bars* 1 μm, 100 nm, and 200 nm

A more detailed analysis of the effect of *B.* *subtilis* SYX04 and SYX20 on fungal cells was performed using TEM (Fig. 4). The hyphae of *M.* *oryzae* P131 without antagonistic bacteria displayed typical eukaryotic cytoplasmic components enclosed by an electron-transparent cell wall (Fig. 4a–c), here indicated with boxes and arrows, observed under TEM. However, mycelia that had been treated with liquid culture of *B.* *subtilis* displayed ultrastructural abnormalities in the hyphal and conidial morphology structure. After exposure to liquid culture of *B.* *subtilis* SYX04, the cell walls of *M.* *oryzae* P131 hyphae appeared rough, and the cytoplasm separated from the cell wall (Fig. 4e), indicated with arrows. The cytoplasm of some conidia separated from cell wall of conidia (Fig. 4d), emphasized with boxes. When hyphae and conidia were exposed to liquid culture of *B.* *subtilis* SYX20, the cellular degradation was severe (Fig. 4f, g) with the presence of indiscernible organelles in the cytoplasm. The cell wall was severely degraded and cytoplasm had exfoliated from the cell wall, indicated with boxes. The co-culture time at 24 h, observation made by TEM indicated that the cytoplasm in conidia had almost completely disappeared and the cell wall had become severely degraded. Such morphological changes were also evident in hyphae.

Overall, the cells of *M.* *oryzae* P131 treated with liquid culture of *B. subtilis* SYX04 and SYX20 exhibited abnormal morphology and cellular disorganization, suggesting that SYX04 and SYX20 degrade the cell wall, destroy the cell membrane, and damage cellular organelles.

### Induction of defense enzymes

**Fig. 5:**
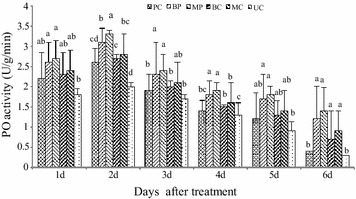
Quantitative changes in plant defense enzymes (PO) in biocontrol of rice blast. Treated with a mixture of fermentation broth of SYX04 and pathogen infection (BP); treated with a mixture of fermentation broth of SYX20 and pathogen infection (MP); treated with a fermentation broth control of SYX04 (BC); treated with a fermentation broth control of SYX20 (MC); treated with a pathogen control (PC); and untreated control (UC). Mean values are based on three replicates. *Bars* represent SD

**Fig. 6:**
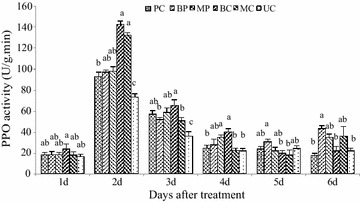
Quantitative changes in plant defense enzymes (PPO) in biocontrol of rice blast. Treated with a mixture of fermentation broth of SYX04 and pathogen infection (BP); treated with a mixture of fermentation broth of SYX20 and pathogen infection (MP); treated with a fermentation broth control of SYX04 (BC); treated with a fermentation broth control of SYX20 (MC); treated with a pathogen control (PC); and untreated control (UC). Mean values are based on three replicates. *Bars* represent SE

**Fig. 7:**
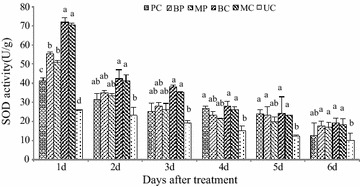
Quantitative changes in plant defense enzymes (SOD) in biocontrol of rice blast. Treated with a mixture of fermentation broth of SYX04 and pathogen infection (BP); treated with a mixture of fermentation broth of SYX20 and pathogen infection (MP); treated with a fermentation broth control of SYX04 (BC); treated with a fermentation broth control of SYX20 (MC); treated with a pathogen control (PC); and untreated control (UC). Mean values are based on three replicates. *Bars* represent SE

The activity of PO increased gradually, peaking at 2 days after inoculation (Fig. 5), showing about a two-fold increase in PO levels. PO levels were significantly different in leaves treated with the mixture compared with those treated with *M. oryzae* or *B. subtilis* alone. However, PPO activity was significantly higher in plants treated with *B.* *subtilis* alone than in all other treatments after 2 days (Fig. 6). The activity on the second day increased three-fold and a gradual decrease was observed on the third day. The highest activity of plants treated with *B.* *subtilis* SYX04 was observed on the second day, and the PPO activity of plants treated with *M.* *oryzae* alone was lower than that of those treated with the mixture of *M.* *oryzae* and *B. subtilis*. The activity decreased on the second day of treatment. As shown in Fig. 7, the activities of superoxide dismutase (SOD) in the rice increased dramatically after treatment with liquid culture of *B.* *subtilis* SYX04 and SYX20 alone, but activity was highest on the first day. The SOD activity of plants treated with the mixture of *M.* *oryzae* and *B. subtilis* was higher than that of those treated with *M.* *oryzae* alone on the first day. PO, PPO and SOD associated with disease resistance characteristics were induced in the rice plants sprayed with *B.* *subtilis* SYX04 and SYX20.

## Discussion

In this research, the interaction between *B.* *subtilis* and rice plants was studied for the purpose of assessing potential biocontrol mechanisms for rice blast. Many reports indicate that *Bacillus* strains are beneficial to stimulation of plant growth and disease resistance. Previously, *B.* *subtilis* strains were used as biological control agents, in which capacity they played an agriculturally important role in biocontrol of several plant pathogens, including *M.* *oryzae* (Zhang et al. 2014; Leelasuphakul et al. 2006). *B.* *subtilis* activity rely on special biological control mechanisms that *Bacillus* spp. can compete for colonization sites in plants, form biofilms, and enhance the provision of nutrients and phytohormones; improve interaction of pathogen and plant; and inhibit growth of the pathogen by antibiotics (Surfactin, Iturin and Fengycin), toxins, and biosurfactants; and a mechanism associated with bacteriolysis, involves production of extracellular cell wall degrading enzymes such as chitinase and β-1,3-glucanase (Chernin and Chet 2002; Li et al. 2014; Whipps 2001).

Better knowledge responsible for successful biological control on the basis of plant-associated antagonists including the complex regulation of disease suppression associated with bacteriolysis and inhibition of growth of the pathogen by antagonists; the dynamics and composition of plant-associated bacterial communities and colonization sites in plants (Normander and Prosser 2000). To investigate the distribution of GFP-tagged *B.* *subtilis* SYX04 and SYX20 in rice plants further, the bacterial populations in the rice plant was monitored. It has been reported that labeled *Bacillus* NB12 could also be detected after 10 days on newly grown rice leaves that had been exposed to *Rhizoctonia solani* (Zhang et al. 2012). *B. methylotrophicus* BC79 could colonize rice leaves. Its population in the leaves (1.65 × 10^8^ CFU/g) was much larger than in the stems (6.78 × 10^7^ CFU/g) or the roots (3.56 × 10^7^ CFU/g) after 10 days of inoculation. The current results differed from those of previous studies of *B.* *subtilis* (Fig. 1). There is little information about colonization of *B.* *subtilis* in rice plant towards *M.* *oryzae*. Due to loss of the GFP-expressing plasmids, the GFP-expressing bacterial population was assayed in rice leaves only 20 days after inoculation. Further research should be performed to detect the bacterial populations of strains SYX04 and SYX20 in rice leaves after one or 2 months of inoculation. They should be labeled with double antibiotics or other markers.

Studies on the interactions between *M.* *oryzae* in rice and *Bacillus* sp., and the mechanisms of in vivo biological control are important to the adoption of adequate control measures for rice blast which are ecologically sustainable. Wang et al. (2012) described the molecular mechanisms involved in *B. cereus*–rice interactions. Xyloglucan endotransglycosylase may be involved in rice cell elongation processes due to up-regulation by treatment with *Bacillus* strains (Jan et al. 2004). The antagonist-pathogen interaction of *B.* *subtilis* KB-1122 and *M.* *oryzae* has been studied using comparative proteome analysis (Zhang et al. 2014). SEM results showed that citral inhibited hyphal growth of *M. oryzae* and reduced spore germination and germ tube length (Li et al. 2014). Taguchi et al. (2003), Jia et al. (2011), and Zhang et al. (2014) also found that hyphal morphology became irregular when the fungus was treated with specific biological control agents. Further research is needed to understand mechanisms of vital antibacterial substance produced by strains SYX04 and SYX20.

TEM results showed that citral caused hyphal morphological and structural alterations (Li et al. 2014). Harish et al. (1998) reported that in vitro interaction of *F.* *udum* and *B.* *subtilis* strain AF 1 formed chlamydospore-like structures and increased vacuolation in the mycelium, when both cultures were simultaneously inoculated in PDB. These observations were consistent with reports showing the degradation of fungal cell walls treated with fermentation culture of *B.* *subtilis* NSRS89-24 (Leelasuphakul et al. 2006). The results showed that spore germination, germ tube length, and appressorium formation were significantly inhibited by liquid culture of SYX04 and SYX20 (Table 1) and a similar trend was observed for ultrastructural abnormalities in the hyphal and conidial morphology (Fig. 4). After exposure to *B.* *subtilis* SYX04 and SYX20, the hyphae displayed partial distortion, severely degraded cell walls and cytoplasm that had exfoliated from the cell wall (Figs. 2, 3). The results indicated that *B.* *subtilis* SYX04 and SYX20 may secrete molecules that mediate the interaction as early as 12 h after incubation. Further studies are necessary for identifying the elicitor molecules during the initial stages of the rice-*M.* *oryzae* interaction.

The defensive capacity was found to be enhanced when a plant was stimulated by biological control agents (van Loon et al. 1998). When rice seedlings were infected by pathogens, the increased activity of various defense-related enzymes and chemicals was found to enhance ISR (Chithrashree et al. 2011). The peroxidase activity in rice leaves infected by *M.* *oryzae* exhibited an increase (Wang et al. 1991; Sena et al. 2013). Peroxidase is related to diverse responses to plant stress. PO can reduce pathogen viability and spreading directly and indirectly because its enzymatic activity is relevant to the production and modulation of active oxygen species (Lamb and Dixon 1997). PPO can terminate oxidation of diseased plant tissue, and its enzymatic activity contributes to its role in disease resistance (Kosuge 1969). Guo and Li (2014) have researched *Paenibacillus kribbensis* PS04 for induction of plant resistance in the control of *R. solani* in rice. The application of a mixture of both *B.* *subtilis* and *M.* *oryzae* resulted in the induction of more PO activity against *M.* *oryzae* in rice plants than *B.* *subtilis* alone (Fig. 5). PPO and SOD activity in plants treated with *B.* *subtilis* alone were significantly higher than in all other treatments after inoculation (Figs. 6, 7). The results presented herein allow us to conclude that *Bacillus* sp. can induce plant defense mechanisms in rice, but the metabolic pathway that is activated need to be studied further.

## Conclusions

*Bacillus* *subtilis* SYX04 and SYX20 were found to significantly inhibit rice pathogen *M.* *oryzae* P131, elicite plant defense reaction, and colonize in rice leave. Strains SYX04 and SYX20 significantly suppressed the mycelial growth, conidial germination, and germ tube formation, and appressorial formation of *M.* *oryzae*. GFP-labeled strains of *B. subtilis* colonized rice mature leaves, reaching populations of more than 5 × 10^6^ CFU/g after 20 days, and labeled strains could be also detected after 3 days on newly grown leaves. After *Bacillus* spp. treatment, expression of defense-related peroxidases (PO, PPO and SOD) increased significantly, and these enzymes may participate in the defense mechanisms of plants. This is the first detailed report of the interaction between GFP-tagged *B.* *subtilis* and rice plant was performed towards controlling of rice blast.
